# Prospective evaluation of the impact of potent antiretroviral therapy on the incidence of Kaposi’s Sarcoma in East Africa: findings from the International Epidemiologic Databases to Evaluate AIDS (IeDEA) Consortium

**DOI:** 10.1186/1750-9378-7-S1-O19

**Published:** 2012-04-19

**Authors:** Jeffrey Martin, Megan Wenger, Naftali Busakhala, Nathan Buziba, Mwebesa Bwana, Winnie Muyindike, Ruth Mbabazi, Erin Amerson, Constantin Yiannoutsos, Beverly Musick, Philip LeBoit, Timothy McCalmont, Beth Ruben, Toby Maurer, Kara Wools-Kaloustian

**Affiliations:** 1 University of California, San Francisco, USA; 2 Moi University, Eldoret, Kenya; 3 Mbarara University of Science and Technology, Mbarara, Uganda; 4 Indiana University, Indianapolis, IN, USA

## Background

Prior to the rollout of potent antiretroviral therapy (ART), Kaposi’s sarcoma (KS) was the most commonly reported malignancy in many resource-limited settings, such as most of sub-Saharan Africa. In resource-replete settings (e.g., U.S. or Europe), the advent of ART—and the availability of established research networks—resulted in documentation of a marked decrease in KS incidence. In contrast, in resource-limiting settings like Africa, the heretofore lack of epidemiologic infrastructure has limited our knowledge about the effect of ART on KS incidence.

## Methods

We prospectively followed HIV-infected adults, without prior KS, attending 26 HIV/AIDS clinics at the Mbarara (Uganda) or AMPATH (Kenya) sites of East Africa IeDEA. Time zero was October 2008, when we introduced skin punch biopsy for KS diagnosis, which was available same-day free of charge. Biopsies were adjudicated by board-certified UCSF dermatopathologists. Patients were followed until they developed KS, death, loss-to-follow-up, or administrative closure. Once started on ART, patients were considered on ART irrespective of adherence/interruptions. Incident KS was defined as any new occurrence of KS 30 days after clinic enrollment that was documented either pathologically or by clinical diagnosis in the absence of pathology.

## Results

We followed 98,024 HIV-infected adults: 31% men, 66% ever on ART, and median values at study enrollment of 35 years old (IQR: 29-43) and 277 CD4+ T-cells/mm^3^ (IQR: 137-453). Patients were followed for 144,182 person-years (median 1.8 years/patient) for 499 incident KS diagnoses, 43% of which were pathologically confirmed. KS incidence during non-ART use was 1876 cases/100,000-person-years in Uganda and 596 in Kenya; incidence during ART use was 201/100,000-person-years in Uganda and 270 in Kenya. After adjustment for age and gender, ART-users had a substantially reduced rate of KS compared to non-users: 88% reduction in Uganda (p<0.001), and 50% in Kenya (p<0.001). Further adjustment by CD4+ count showed a persistent ART effect, suggesting ART benefits for KS prevention above and beyond CD4 restoration (Table). In ART-users who achieved a CD4+ count of >350 cells/mm^3^, KS incidence declined to 18/100,000-person-years in Uganda and 93/100,000-person-years in Kenya.

**Table 1:** 

KS Incidence During Non-ART Use and ART Use in East African HIV-infected Adults						
	During Time of No ART Use			During ART Use		

CD4+ T cell count (cells/mm^3^)	No. KS cases	Person-years	Rate (95% CI)*	No. KS cases	Person-years	Rate (95% CI)*

0-50	19	645	2943 (1877, 4615)	62	4070	1523 (1187, 1954)
51-100	2	613	326 (81, 1304)	37	5166	716 (518, 988)
101-200	8	1682	475 (237, 951)	45	15651	287 (214, 385)
201-350	27	6383	423 (290, 616)	40	27035	147 (108, 201)
>350	23	13926	165 (109, 248)	31	37804	82 (57, 116)

* per 100,000 person-years

## Conclusions

In a prospective study in East Africa, KS incidence was very high in untreated HIV-infected adults but was substantially reduced by ART — similar to that observed in resource-replete settings. Despite ART, absolute rates of KS remained considerable until a CD4+ count of >350 was achieved, suggesting the need for earlier ART initiation. The IeDEA platform provides unique opportunities for prospective African KS research.

